# Patriotism and the Nurse-Training Schools

**Published:** 1915-11-20

**Authors:** 


### PATRIOTISM & THE NURSE-TRAINING SCHOOLS.

### IV. SOME LONDON POOR-LAW INFIRMARIES.

In no training schools have so great efforts been made for the wounded as in those attached to the Poor-Law hospitals. In some of these institutions, owing to the fact that they have been commandeered by the War Office, the training schools are actually in abeyance. In others, a double hospital is being carried on, the sick poor being nursed in one building and another being occupied by wounded or sick soldiers. When, in addition to such radical alterations, the nursing staff has been depleted in the interests of military and naval hospitals at home and abroad, it will appear that uncommon difficulties have beset the path of those responsible for maintaining a high standard of nursing in these great public establishments. A few examples will serve to illustrate how these difficulties are being surmounted.

### Edmonton Military Hospital.

This infirmary was taken over by the Army Council as a war hospital in May 1915, together with the *personnel*. To the credit of the matron be it recorded that the training school is still carried on. The civil patients were removed to the old infirmary, and the probationers continue their training under the matron's supervision, being interchanged between the military hospital and the infirmary. The ward sisters and other administrative officers are permanently established in either the military hospital or infirmary to which they are appointed. As all this happened some months after the war broke out, and before the pressing demands of the institution in regard to nursing the wounded within its own walls were manifested, several of the staff were called up for duty in military hospitals. Two of these have already returned to the military branch of their own institution. All the probationers who are completing their full course of training are being engaged as staff nurses in the military hospital.

### Hampstead Infirmary.

The infirmary and workhouse at Hampstead were both taken over by the War Office in the spring, and they now constitute a military hospital of between 400 and 500 beds. The entire staff has remained on under the altered conditions.

### Marylebone Infirmary.

No fewer than eighty St. Marylebone Infirmary trained nurses, of whom we append a list, are at present occupied in military nursing at home and abroad. Some idea of the strenuous work accomplished by the matron, Miss Cockrell, is gained by the simple announcement that nearly all the ward sisters have gone off to do military work. Their posts have been kept open for them, in case any may desire to return, but a few resigned at the end of the first year of war, having signed on for another year in their respective services. Two members of the training school have met with distinction. Sister A. H. Ivin was mentioned in dispatches June 1915, and shortly afterwards received the Royal Red Cross from the hands of the King at Buckingham Palace. She was trained at Marylebone, and was successively ward sister, theatre sister, night superintendent, and store-room sister. From the latter post she was called up for war duty in August 1914, and since last October she has been in France. Nurse Dorothy M. Best, who was mentioned in Sir John French's dispatches in June last, has been in France since the beginning of the war, and has lately been transferred to Aldershot.

The following nurses trained at this infirmary are on active service:—

*Military Hospitals in Great Britain and Ireland.*—Sisters Fishwick, Gribble, Moxon, Pomeroy, Tiplady, Parker, McLean; Nurses Bucumshaw, Boniface, Bowles, Barrett, Betterill, Collins, Cutler, Crawford, Downing, Everett, Eckstein, Fairweather, Gladwin, Gray, Green, Jack, Jones, Livingstone, Little, Lambert, McSweeney, Nicol, Norman, Pain, Poole, Pepper, Potter, Pellowe, Roberts, Storey, Stone, Sinclair, Sainty, Turner, Tomkins, Tull, Woolcot, Whissell, Winward, Wakeford, Welton. Miss Macmillan is matron of the Borough Hospital, Birkenhead (a hundred beds for wounded), and Miss Parkes is assistant matron at the military hospital, Dublin Castle. *Q.A.I.M.N.S.*—Sisters Denne (Flanders), McCormack, and Toller. *Hospital Ships.*—Sisters Bicks, Cameron, Farmer, and Watt. *France and Flanders.*—Nurses Kyte, Park, and Rask. *Egypt.*—Nurses Aland, Bishop, Joy, Macklin, Paterson, Ivy Smith, and Wilson. *Serbia.*—Nurses Giles (roadside dispensary), Pritchard, and Scott. *Mediterranean.*—Nurse Adair. *British Red Cross.*—Nurses Buckingham and Humphreys. A record of which any institution might be proud.

### Whitechapel Infirmary.

The following members of the training school have left the staff since the war began to take up war duty, at home and abroad:—

Miss Eleanor A. G. Taylor, assistant matron, has gone to the 4th General Hospital, King's College. Sister E. Laurie is at the military hospital, Colchester. Staff-nurse A. Aylward left for Pembroke Docks. Staff-nurses A. Deverell and A. Binnie are at stationary hospitals in France. Staff-nurses A. Golding, L. Phillips, K. Coombes, and J. Kingsford are working in Territorial hospitals in England. Staff-nurses E. Taylor and Mrs. Jury are working in Red Cross hospitals. The following who were trained in the Whitechapel Infirmary are also serving in various localities on war service:—Miss Grace Preston, matron; Sisters L. Rae, M. Brown, E. Foskett, S. Roberts, M. Ready, E. Loud. Staff-nurses B. Dodd, M. Jones, E. Bardley, E. Jones, L. Smith, Matthews, Vincent, and Mrs. Hatch.

